# 110 Sleep After Discharge: A Cohort Study of Burn Participants in a Home-Based Virtual Rehabilitation Trial

**DOI:** 10.1093/jbcr/irad045.083

**Published:** 2023-05-15

**Authors:** Lori Rhodes, Stephen Sibbett, Caitlin Orton, Gretchen Carrougher, Barclay Stewart, Aaron Bunnell, Tam Pham

**Affiliations:** Harborview Medical Center, Seattle, Washington; University of Washington, Seattle, Washington; University of Washington, Seattle, Washington; University of Washington, Seattle, Washington; University of Washington, Seattle, Washington; University of Washington, Seattle, Washington; University of Washington, Seattle, Washington; Harborview Medical Center, Seattle, Washington; University of Washington, Seattle, Washington; University of Washington, Seattle, Washington; University of Washington, Seattle, Washington; University of Washington, Seattle, Washington; University of Washington, Seattle, Washington; University of Washington, Seattle, Washington; Harborview Medical Center, Seattle, Washington; University of Washington, Seattle, Washington; University of Washington, Seattle, Washington; University of Washington, Seattle, Washington; University of Washington, Seattle, Washington; University of Washington, Seattle, Washington; University of Washington, Seattle, Washington; Harborview Medical Center, Seattle, Washington; University of Washington, Seattle, Washington; University of Washington, Seattle, Washington; University of Washington, Seattle, Washington; University of Washington, Seattle, Washington; University of Washington, Seattle, Washington; University of Washington, Seattle, Washington; Harborview Medical Center, Seattle, Washington; University of Washington, Seattle, Washington; University of Washington, Seattle, Washington; University of Washington, Seattle, Washington; University of Washington, Seattle, Washington; University of Washington, Seattle, Washington; University of Washington, Seattle, Washington; Harborview Medical Center, Seattle, Washington; University of Washington, Seattle, Washington; University of Washington, Seattle, Washington; University of Washington, Seattle, Washington; University of Washington, Seattle, Washington; University of Washington, Seattle, Washington; University of Washington, Seattle, Washington

## Abstract

**Introduction:**

Patients with major burns experience physiologic changes that can impact their recovery for months after hospital discharge. Little is known about sleep quantity and quality in the post-discharge period. Here, we report on actigraphy sleep data for participants enrolled in a prospective trial of home-based virtual rehabilitation.

**Methods:**

We conducted a randomized controlled trial to evaluate the effect of home-based rehabilitation using Jintronix® modules and Kinect® motion sensor on adherence to therapy. In both intervention and control (usual care) groups, participants were provided a wrist actigraphy accelerometer device (Vivofit®) to wear for the 12-weeks intervention. Sleep data were retrieved remotely and analyzed. Actigraphy data were defined as complete and analyzable if participants had 5 out of 7 days of actigraphy wear and upload in a week. Average weekly sleep was calculated and reported by group assignment. Descriptive statistics were used for comparisons, with p< 0.05 deemed as significant.

**Results:**

Forty-four participants were enrolled with a 1:1 group assignment. Mean age was 38.5 years (SD=14.8), mean TBSA was 15.6% and most were male (68%). Average sleep was well within healthy population norms, with little difference between intervention and controls (7.29 vs.7.20 hours respectively, p=0.25) in the 90 days after discharge. Participants in control group spent more time in light sleep (4.05 vs. 3.88 hours, p< 0.01). The percent deep sleep, however, was comparable to healthy population norms (2.92 and 2.75 respectively, p=0.25). Participants in the control group experienced a higher proportion of nights with sleep disturbances (0.75 vs. 0.70, p=0.006).

**Conclusions:**

Remote wrist wearable-wearable actigraphy data provide an important glimpse into sleep duration and quality for burn injured individuals in the 12 weeks after hospital discharge. An encouraging finding was that total sleep and deep sleep proportion for this cohort were comparable to healthy population norms. Though intervention and control groups were similar in total sleep hours, there were important differences in sleep quality.

**Applicability of Research to Practice:**

Remote data acquisition on post-discharge sleep is feasible.

